# Integration of Healthcare Clowns into Pediatric Palliative Care: A Bridge Between Life and Death

**DOI:** 10.5334/ijic.6506

**Published:** 2022-11-15

**Authors:** Victoria Valdebenito Mac Farlane

**Affiliations:** 1 School of Psychology, Universidad Adolfo Ibáñez, Chile

**Keywords:** healthcare clown, pediatric palliative care, death

## Abstract

**Introduction:**

The objective of this paper was to describe the vision of death from the perspective of families of children who experienced palliative care, and team members working in one unit, and to explore the roles of healthcare clowns in working with life and death. The major research of which this paper is part was a requirement of one healthcare clown organization, that since 2008 works as members of the palliative care unit in a public hospital in Chile.

**Description:**

Using a qualitative methodology, and an emergent and descriptive design, 26 people, including mothers and team members of one palliative care unit, participated in in-depth interviews and discussion groups separately. Data analysis was performed using grounded theory and critical discourse analysis techniques.

**Results:**

The roles played by healthcare clowns in palliative care were accompanying, mediating between team members and families, facilitating to process death, provision of humane care using socioemotional competences, promotion of social relationships, and being complementary therapy.

**Conclusion:**

The six roles of healthcare clowns identified by this research have implications for public policies and actions in palliative care. There is also a need to expand this type of work to other public health services in Chile.

## Introduction

Pediatric palliative care (PC) are services that help children suffering from serious illnesses and who are considered untreatable, to prevent or manage symptoms and side effects, both from the disease and from treatments. Its purpose is to help people to feel better [1]. PC also covers emotional, social, practical and spiritual aspects of the disease, thus delivering a better quality of life to people in this period.

On the other hand, healthcare clowns (HC) are interdisciplinary professionals trained in the technique of clowning and in in-hospital regulations [2]. Its main objective is to contribute to the well-being of patients, families and workers using fantasy, play, games and other artistic techniques, being a type of complementary and alternative medicine [3]. In the context of pediatric oncology PC, HC are perceived as a mediator figure, highly valued as complementary therapy [4].

Inseparably, death is linked to PC, leaving families and close friends of those in this category with their lives shaken. That is why PC work should contemplate approaching mourning since talking about death is still a taboo in many societies, even more in children [5]. Internationally, every year more than one million children under the age of 15 require PCs, of which 98% live in low and middle-income countries [6].

In Chile, nearly 520 children annually suffer from oncological disease [7]. Of these, approximately 85% are cared for in the public health system. Equally, this is the second most common cause of death among children. Since 2003, a multi-pronged care strategy has been in place, achieving recovery in more than 70% of these cases. However, development of specific PC programs for children with other conditions is still a big challenge given that all are treated in oncology units of pediatric hospitals. Moreover, there is evidence that indicates that children are excluded as relevant social actors in health discourse and lack of protection of their rights [8], unlike what happens in other countries, such as Argentina [9].

Knowledge about death being close has different emotional effects [10] on people. Specifically, grief over the death of a child has characteristics because of the value of the bond between parents and children. Such loss destabilizes identities, generates pain and total bewilderment, which can trigger the loss of expectations about the future [11].

There is also evidence which indicates that each family members’ experience with death has a direct influence on the development and overcoming of grief, both at individual and family level [12,13]. That is why the approach to mourning should be a personalized and sensitive work based on the needs of the different family structures. Regarding this, some point to the importance of health services becoming aware of and promoting preventive work [12] before the death of the child, including interdisciplinary services of professionals well prepared to do this work [12]. The death of a child also generates changes in family dynamics; grief affects these relationships and changes them [13].

All the above points to the need for further training in PC and in coping mechanisms to face death, suggesting the inclusion of specific training of this kind in medical and health schools [14,15], in favor of promoting more integrated care [16].

Since the emergence of HC programs during the 1980s in North America, their acceptance as valuable members of interdisciplinary health teams has been increasing. Considered as an integral part of care delivery processes [17,18], HCs are also considered agents who add a human dimension with their presence to the work in hospitals [19]. However, despite the increase of HC programs, there is still a lack of research on their effects in PC [20,21,22,23]. A study in Spain on its presence in an oncology room [24] showed positive results in this context. There is also positive evidence on training PC nurses in complementary therapies [25]. However, further research is needed.

Some have compared HCs to shamans and traditional healers [26], playing different roles in society, not only entertaining and making laugh [27]. Thus, their work is deeper, validating the entire range of human emotions. In health contexts, for example, these professionals promote awareness about humane care, thereby claiming human rights [28] and playing a political role through arts [29].

Moreover, previous evidence indicates that HCs have a positive effect in psychological [30,31] and physical [31] symptoms faced by PC oncological patients, improving their quality of life and social support [32].

## Problem statement

In 2008, a HC organization integrated a PC unit interdisciplinary team, composed of an oncologist, who coordinates the team, two nurses, two psychologists, two social workers, an occupational therapist and two professional HCs. They meet once a week to organize work with each child according to their needs. Depending on the case and diagnosis, they visit patients within the hospital, having also available a free home visits service. HC is available for all patients referred to this pediatric PC unit. This is a unique case in Chile and the results presented here part of the first research in Chile about HC in PC.

The PC unit is located in a public hospital in a vulnerable socioeconomic area of Santiago, Chile. The present study was a requirement of the HC organization interested in collecting evidence about the qualitative impacts of its work since joining the unit. They work using the clown as an instrument and their main language.

The major research of which this paper is part was developed between December 2019 and December 2020, and its main objective was to understand the roles played by HC in PC and its qualitative impacts. Specifically, this paper addresses the objective of describing death from the perspective of the children’s family and PC unit team members, and the perceived roles HCs play in such context.

## Ethical approval

Two ethic committees approved this study: one of the hospital (reference n°2886) and one from a university (reference n°59/2019).

## Description of the care practice

### Study design

HC programs first appeared in Chile in 2007. The studied unit is part of the public health system, funded with general taxes, compulsory contributions, and co-payments made to the National Health Fund. At the same time, a large part of the most socioeconomic disadvantaged population, a vast majority, uses public health [33,34]. There is no budget in these institutions to incorporate complementary medicines such as HC. In this case, the HC program is funded by the city hall where the hospital is located, being the only case in Chile of pediatric PC which includes HC.

An interpretivist paradigm underpinned the study. A qualitative strategy was considered appropriate due to the research focus on people’s perspectives on the roles of HCs in PC, with an emerging and descriptive design.

### Data collection

Two techniques were used for data collection: in-depth interviews, and discussion groups with mothers and PC team members separately. Interviews were conducted by the main researcher, who had no previous relationship with participants. An informed consent document was signed by participants before the interviews. The process started face-to-face in locations which suited them, but had to go online to the Zoom platform, due to covid-19 restrictions, while discussions groups were held live when restrictions were lifted. Both, interviews and discussion groups were audio recorded, lasted one hour and a half approximately, and were no repeated. Afterwards recordings were transcribed.

The sample is composed of sixteen mothers of children who passed the PC experience and ten team members of the PC unit, with no dropouts. A successive strategy was used to reach the sample, while a point of saturation principle determined the number of participants. Inclusion criteria were, for families to be have lost children in PC, being over 18 years, and for staff to have work in the unit for a minimum of two years. Exclusion criteria were being under age, and being diagnose with a psychopathology.

### Data analysis

Data was analyzed with critical discourse analysis [35] and grounded theory [36], coded by two researchers. No software was used. Themes derived from data. The coding process was organized according to grounded theory, firstly performing an open coding, then an axial coding and finally a selective coding process. Subsequently, a triangulation process was developed. Participants provided feedback of the findings.

Quality of the research was assured through credibility, auditability and transferability criteria [37], and strict ethical criteria such as confidentiality. Likewise, interviewees are quoted using a parenthesis denoting the number of interviews assigned and fictitious names to protect anonymity. Quotes were selected due to their representativeness of the thematic domain.

Also, as it is a quantitative study, the positionality of the researcher must be informed. Besides of her career as a Ph.D and researcher, she has work as a healthcare clown in different countries. However, she has not work in the studied PC unit.

## Results and analysis

### Caractheristic of study sample

Out of the total sample, family members were sixteen mothers, whom identified themselves as women, whose ages ranged from 29 to 65, most of them married, and all living in the southern part of Santiago. For their part, the ten PC unit team members identified themselves all as women, aged 29 to 63, all holding a university degree, and most of them being single at the time of the study.

As expected, there is a perspective of the PC period as painful and complex one. As a result, a distance between team members and families can be experienced, and a rejection of the latter to biomedical professionals. In this context, HC’s plays a mediating role. To refer to this function, some use the metaphor of a ‘bridge’, like the following participant:

*“Clowns started as a support and were included in meetings little by little because they had a relationship with parents and children that was quite different from ours. It is a very strange situation because clowns are part of the team and they are acting as a “bridge”. They are seen as an entity that does not belong to the team directly. Clowns are obviously part of the health team, and they participate in decision-making, planning what to do about children in palliative care. But families see them as part of the hospital, but not part of the team. So, it’s good for one side but, being a health professional involves other aspects such as to poke them, place them the catheter, do procedures, and give parents and kids the bad news. Therefore, you obviously must stand up and face what is happening, whether you like it or not. As much as you put all the care and affection in it.” (S1)*

Coupled with this, they play an accompanying role. There is a perception that HC is a positive aspect of the entire PC process. Most participants say that HCs are the only positive reminiscence of that time. Commenting on this, one person says:

*“They helped me see that everything was not bad. They arrived at the worst moment of my life, at the worst of the worst. They are the sweeter part of the bitterest moment of my life. They were the ones who managed to return me to that place without feeling anguish or reliving it. I return to those moments with those memories, with the tranquility that you can only have with them.” (M10)*

Moreover, HC plays a role facilitating elaboration about death. It is important to note that this PC unit also works with bereaved families after the child’s death. The HC organization has a physical space within the hospital, which has trees that families have placed there when their children have left.

Many participants believe that such effect on memories is a result of the competences that HCs deploy. One such skill is empathy. Referring to this, a mother points out:

*“They put themselves in the place of the other person. They have that ability. They could put a barrier in this role and pretend like nothing happened. You realize it is sincere; it is born from the heart, not a predisposed thing. Clowns are somewhat intuitive in that sense. In other words, within empathy they have developed an intuition to capture the needs of each child, of each mother. You have to let your heart open to have the ability to understand others.” (M4)*

There is consensus among participants on the high value of empathy within PC.

On the other hand, despite having gone through the PC experience, according to team members there is a uniqueness in the perceptions about life and the death process for each person, and HCs respect that, working according to the person’s needs. About this, one participant explains:

*“Each child understands death according to their age. Also, that understanding is determined by family beliefs. The relationship with death is linked to how those who are caring feel. For example, sometimes making the family feel good is a priority for children. I believe that when we validate their emotions, their fear, their anguish, their grief, they can talk about death, about what’s going to happen when they’re not here. Everything varies with age and the family, how prepared or not they are to talk about death. Here clowns have been a contribution, being a support to be able to work together with children and their families.” (S8)*

Likewise, as part of their accompaniment work in PC, in some cases, families invite the HC to the moment of transition from life to death of their children, leading a ritual for those experiencing loss, applying emotional intelligence in the form of respect and sensitivity. One mother relates her experience by saying:

*“The doctor told me: “She stopped breathing, she’s gone”. I went down looking for the clowns, looking for their smile, their friendship, the person who was going to hug me and tell me what I was going to do, what was going to become of my life. Because when the doctor told me that, I wanted her to tell me that I was gone too. A part of me went with her. I didn’t understand what was going to happen from there; I didn’t understand how I was going to continue my life, even though I had a baby of months with me. When the clown arrived, she saw me from the street coming down and she dropped everything and hugged me, and that was what I needed. So, then I told her I wanted them to be there and they came up. They sang songs that my daughter loved. The clowns came up to sing, to hug us, in between the songs. Juliet left with so much love, with everything she wanted, there was everyone she wanted at her farewell.” (M5)*

Also, HC promotes social relationships. For some mothers, the bond established with the HC is fundamental to face not only this stage but also the departure of their children. Referring to the power of this bond, a team member comments:

*“I think what they value the most is unconditionally of the clowns, the fact that they are there with them. No matter what time it is, they know that they will always be available to listen to them, to be with them: and not only in the role that one thinks a clown has, of joy. On the contrary, also in sadness, anger.” (S5)*

Thus, according to the participants, the HC with his competences, and validating all emotions, delivers humane care and allows them to talk about death. HCs work with death, humanizing health contexts. In this regard, another participant adds:

*“One of the things we did at the beginning of working with them [HCs] was to define working days to get to know each other as people, where we didn’t talk about medicine or procedures, but about who is who, what are their fears, what are their experiences with death, what is death for each one. I know the clowns are very capable in that area and I can ask them help with that. That’s why I tell you that death is seen from the person, not from the clown. Death summons us as people, with the resources we have, with our stories, with our defects that we all have.” (S2)*

At the same time, many participants perceive the HC as a figure which provides a complementary therapy, making the work in PC integral and holistic. Likewise, according to some discourses, the presence of HC in this PC unit is a very different reality from many others in public hospitals in Chile.

## Discussion section

### Main results and implications

According to these results, HC accompanies children and families in PC, mediating and facilitating social relationships, being a bridge of magic and fantasy [38]. In addition to the evidence on its positive psychological and physiological effects on cancer patients [30,31,39], these results are in line with evidence showing that it has social effects, for example on the organizational climate of the hospital [40,41], and on the quality of life and social support of PC patients and families [32].

In the roles of mediation and processing death, HCs are a kind of shaman [26], and evidence has already highlighted the relevance of shamanic mythology in the context of PC [42]. Here, assertive communication skills are also central. This contributes to talking about death with children, dissolving the taboo [5].

Particularly in PC, HCs deliver a holistic treatment that complements [4,43,44], based on each person’s needs, in line with what is suggested [10]. They also generate positive memories of the process consistent with international evidence [45]. These findings also resonate with research indicating that, in working with death, spiritual aspects are fundamental [46].

Alongside all these consensuses, there is a diversity of perspectives on death. These are linked to social factors such as family values, other contextual factors and the child’s stage of development [47,48], being social constructs [49].

On the other hand, it emerges from this study that one of the HC’s roles is to deliver humane care, promoted by the Chilean Ministry of Health in the last decade. Humane care is a reaction against the possible dehumanization of health care due to various structural changes, new models and technologies. Faced with this, this approach invites professionals to rescue human, spiritual and transpersonal aspects [50]. The HC is able to work on death given that it incorporates these human characteristics [27] (Table 1).

**Table 1:** Thematic Analysis.


ROLES/AGENTS	MOTHERS	STAFF

**i. Mediating role**	x	x

**ii. Accompaniment**	x	

**iii. Facilitates to process death**	x	

**iv. Promotion of social relationships**	x	x

**v. Provision of humane care using socioemotional competences**	x	x

**vi. Complementary therapy**	x	x


Related with humane care are socioemotional competences [51]. One of such, which HC put into play, is empathy, part of emotional intelligence [52], a construct that combines cognitive aspects, such as recognizing and identifying with the feelings of another person, and emotional elements when experiencing such feelings [52].

Likewise, socioemotional competences are considered fundamental for 21st century professionals [53], which further highlights the relevance of including this in healthcare curriculums. Likewise, in making decisions about health care, especially in pediatric PC, professionals need to listen to children and families’ perspectives [9].

These results represent one PC unit that is part of a public hospital in Chile, which as such reflects huge inequalities in compare with the private system [54]. The unit is also located in an area with a multidimensional poverty rate of 27.11%, higher than the average of Santiago (15.01%) and the national average (16.63%) [55], which indicates that the users of this service are among the most disadvantaged of the country. Thus, the work done by the HC also covers social justice, community, identity and memory aspects. This adds a political connotation to HC, working with the most socioeconomic disadvantaged people in one of the hardest life situations.

## Conclusion

The present study has shown six roles of HCs in PC, namely accompaniment, mediation, processing death, favouring social relationships, being a complementary therapy, and providing humane care. Thus, its inclusion in PC provides integrated care.

### Lessons learned

One of the main limitations of the research is the lack of children’ voices. More research about it is then required. In addition, further inquiry in other type of units is needed. As it happens with other evidence about HC, the results presented here have implications for public policy and in developing future HC training programs in Chile, a field that urges research in that setting.

However, the results of the major research were used by the organization to define a training program for HCs specifically working in PC, to improve its work. Equally, the results were used to improve the accompaniment work with grieving mothers. The integration of the work done by this HC organization in PC is unique, both in the context of public hospitals in Chile and within the same hospital. Finally, these results stress the need to spread HC to more public health services in Chile, to provide a better support to those disadvantage people suffering by the loss of their children, a mission that the HC organization has taken.
